# Role of PPAR*α* in the Control of Torpor through FGF21-NPY Pathway: From Circadian Clock to Seasonal Change in Mammals

**DOI:** 10.1155/2009/412949

**Published:** 2009-06-07

**Authors:** Norio Ishida

**Affiliations:** ^1^Clock Cell Biology, Department of Biological Resources and Functions, National Institute of Advanced Industrial Science and Technology (AIST), 6-5 Tsukuba Center, 1-1 Higashi, Tsukuba, 305-8566, Japan; ^2^Graduate School of Life and Environmental Sciences, University of Tsukuba, Tsukuba, Ibaraki 305-8502, Japan

## Abstract

In nature, hibernating animals encounter fasting, cold temperature and short day seasonally. Torpor is a state of decreased physiological activity in an animal, usually characterized by a reduced body temperature and rate of metabolism to adapt such a severe environment. Ablation of the central clock synchronizer, the suprachiasmatic nucleus in brain, abolishes torpor, a hibernation-like state, implicating the circadian clock involved in this seasonal change. Biologists knows well the energy source of daily heterotherms/hibernators changed from glucose to lipids in winter. Here we review several lines of evidence of a master transcriptional regulator in lipid catabolism, PPAR*α*, in the control of torpor through FGF21-NPY pathway. This indicate the importance of circadian—and photoperiod—regulation of PPAR*α* to tell seasons in our body.

## 1. Introduction

Peroxisome proliferator-activated receptor a (PPAR*α*) is a nuclear receptor that regulates the transcription of numerous genes involved in lipid metabolism and energy homeostasis (1,2). Binding of ligands such as fatty acids or fibrate to PPAR*α* leads to obligate heterodimerization with 9-cis retinoic acid receptor (RXR) and subsequent recruitment of coactivators, resulting in the initiation of DNA transcription of target genes [1]. Recent studies have revealed that PPAR*α*-induced fibroblast growth factor 21 (FGF21) plays an important role in adaptation to fasting, such as adipose lipolysis, hepatic ketogenesis and torpor [2–6]. Torpor is often used to help animals survive during periods of winter, and it considered as the state in between sleep and hibernation. In torpor, it is well kown that the energy source of daily heterotherms changed from glucose to lipids in winter. Taken together, FGF21 might be a key regulator for torpor in mice [5]. The metabolic regulator FGF21 has antidiabetic properties in animal models of diabetes and obesity. Recent papers showed that the hepatic gene expression of FGF21 is regulated by PPAR*α* [2–4]. Fasting or treatment of mice with the PPAR*α* agonist Wy-14,643 induced FGF21 mRNA by ca.10-fold [4]. In contrast, FGF21 mRNA was low in PPAR*α* deficient mice, and fasting or treatment with Wy-14,643 did not induce FGF21. Obese ob/ob mice, known to have increased PPAR*α* levels, displayed 12-fold increased hepatic FGF21 mRNA levels. The importance of PPAR*α* for FGF21 expression even in human liver was also shown by Wy-14,643 induction of FGF21 mRNA in human primary hepatocytes. Furthermore, PPAR*α* response elements were identified in both the human and mouse FGF21 promoters [4]. At least in mice, within 1.5 kb upstream region, two putative PPAR*α* response elements were detected by using promoter-deletion assay and chromatin immunoprecipitaton [2]. 

**Figure 1:**
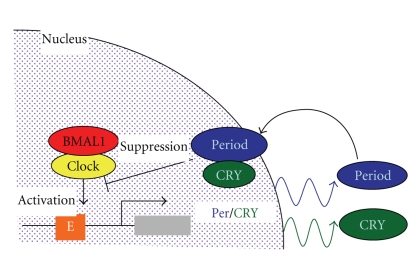
Negative transcriptional feedback loop for 24 hrs rhythm generation in manmals. The circadian clock positive transcription factor, CLOCK and BMAL1 transactivates other negative limbs of clock genes such as *period (per)*, *cryptochrome (cry)* via cis-element E-box in their promoters. These negative components PER and CRY, in turn, suppress the activity of CLOCK/Bmal during about 24 hrs.

The behavior and physiology from Drosophila to man are subject to circadian, 24-hour rhythmicity. Recent molecular studies show that circadian clock are controlled by negative feedback loops in clock gene expression. The circadian clock, an endogenous cell-autonomous machinery of rhythmically acting transcriptional and translational feedback loop. The circadian clock positive transcription factor, CLOCK and BMAL1 transactivates other negative limbs of clock genes such as *period (per)*, *cryptochrome (cry)* via cis-element E-box in their promoters (Figure 1) [7–9]. These negative components PER and CRY, in turn, suppress the activity of CLOCK/Bmal. At least eleven clock genes (clock, period1/2, Bmal1, cry1/2, E4BP4, b-TrCP1/2 casein kinase1 and glycogen synthase kinase 3b) generate circadian rhythms in mammals, where most of these genes were identified as homologues from *Drosophila* clock genes except for Clock [10]. Molecules generally of transcription factors that oscillate over a 24-hour period control their own expression in a circadian fashion seem critical to the generation of circadian rhythm of most organisms [7, 9]. However, except for transcriptional proteins, some kinases that are involved in these transcrptional protein degradation (casein kinase 1 and b-TrCPs ) and nuclear translocation (glycogen synthase kinase 3b) are also important for fine-tuning the concentration of clock molecules at specific subcellular sites [9, 10].

The suprachiasmatic nucleus (SCN) of the anterior hypothalamus in mammals is the central oscillator that shynchronized by stimuli such as light in mammals [7, 9]. In contrast, regular mealtimes induce food-anticipatory behavioral rhythms with circadian properties similar to those of light-entrainable rhythms [11–13] and set the phase of circadian oscillators in most peripheral organs. Interestingly, these food-entrainable oscillator (FEO) did not require SCN suggesting that FEO is the other brain region except for SCN or peripheral tissues. Other peripheral tissues are also equipped with endogenous oscillators using clock gene products. In general, the expression of mRNA and proteins of mammalian clock genes in the SCN as well as other peripheral tissues oscillates in a robust circadian manner Thus, the oscillation of such clock genes is a useful marker for determining the phase and period of peripheral clocks and the central clock(SCN) [14, 15]. Only recently the importance of circadian system has become apparent for the regulation of lipid metabolism, especially for rhythmic PPAR*α* expression because of a convergence of data from microarray analysis using clock mutants animals [10, 16]. Furthermore, a role of clock gene products is considered for stress sensor for cold, short day and no-food in winter. In this review, I will summarize recent progress whether PPAR*α* plays a significant role in regulating specific metabolic pathways strongly associated with torpor/circadian rhythm.

## 2. Circadian Expression of PPAR*α* through Clock Gene Products

PPAR*α* (peroxisome-proliferator-activated receptor *α*) is a member of the nuclear receptor superfamily and it plays a central role in fatty acids oxidation [6, 17]. Interestingly, fatty acids can function as endogenous ligand for PPAR*α*. Hepatic PPAR*α* is expressed in a circadian manner at the mRNA and protein levels in rats [18] and in mice [19]. The circadian expression of PPAR*α* was thought to be regulated by unknown transcriptional molecular mechanism.

But the recent study examined the expression profile of *PPAR* *α* in *Clock* mutant mice that are deficient for the circadian clock for locomotor activity. This showed that the circadian expression of PPAR*α* was dampened in *Clock* mutant mice, because the circadian expression of PPAR*α* is regulated directly by clock gene product CLOCK protein via the E-box-rich region in the second intron of PPAR*α* *in vivo* and *in vitro* [20]. The periodicity of homozygous *Clock* mutant mouse behaviour [21, 22] and body temperature [23] is abnormally long. As the *Clock* allele is truncated and causes a deletion of 51 amino acids, the mutation presumably would not have a significant effect on protei-protein binding domains, leaving CLOCK dimerization and DNA binding intact. The mutant CLOCK protein can still form heterodimers with BMAL1 that bind to DNA, but these are deficient in transactivation [7, 16]. The results suggest that CLOCK/Bmal play an important role in lipid oxidation during the specific time of a day by regulating the circadian transactivation of PPAR*α* in mice. To further support this notion, Turek et al showed that *Clock* mutant mice become obese and developed dyslipidemia [24]. We also showed that leptin deficient obese mice ob/ob with *Clock homozygous *mutantation gained body weight, total cholesterol and triglyceride through adipocyte hypertrophy. These obese phenotype might be explained partly because of dampening for the circadian expression of PPAR [20, 25].

## 3. Circadian FGF21 Induction through PPAR*α* Activation in Liver

Fasting remarkably increases hepatic FGF21 mRNA levels due to PPAR*α* activation via PPAR binding elements in the FGF21 promoter [2, 4]. Within 22 members of fibroblast growth factor (FGF) family, FGF21 was classified as metabolic hormones in the FGF19 subfamily, which comprises FGF19 (mouse FGF15), FGF21 and FGF23 [6, 26]. These FGFs are distinguished from other FGFs in that they function in an endocrine fashion presumably due to having low affinity for heparin. Notably, FGF21 reported to enter into the brain through the blood-brain barrier from liver and kidney [27]. We recently showed that administering mice with bezafibrate, a PPAR*α* ligand, induces time-dependent torpor-like phenomena, decreased body temperature during the latter half of the dark period as explained precisely in the next chapter [28]. The PPAR*α*-FGF21 pathway could be involved in these torpor-like phenomena, because body temperature decreases with time-dependent manner in fasting FGF21 transgenic mice and in the fasting mice infected with an adenovirus expressing FGF21 [2, 5]. However, the mechanism underlying the time-dependent torpor-like phenomena induced by the PPAR*α*-FGF21 pathway has not been elucidated. We also found that FGF21 expression is regulated by a circadian mechanism in mice administered with bezafibrate [29]. We found that a nighttime injection of bezafibrate, a ligand of PPAR*α*, effectively induced FGF21 expression, whereas a daytime injection did not affect it. Furthermore, bezafibrate-induced circadian FGF21 expression was abolished in PPAR*α*-knock out mice. These observations suggest that bezafibrate-induced circadian FGF21 expression is due to circadian variations in the responsiveness of the PPAR*α* system in the liver of laboratory mice.

## 4. Torpor and NPY Induction by PPAR*α* Agonist in Laboratory Mice

Torpor is a state of decreased physiological activity in an animal, usually characterized by a reduced body temperature and rate of metabolism [5, 30]. During the active part of their day, these animals maintain normal body temperature and activity levels, but their body temperature drops during the latter half of dark phase. Torpor is often used to help animals survive during periods of winter season, and it considered as the state in between sleep and hibernation. Animals like hummingbirds, bats and hamsters show torpor. Siberian hamsters (Phodopus sungorus) undergo daily torpor during which body temperature decreases by as much as 20 C degrees and provides a significant savings in energy expenditure [30]. Natural torpor in this species is normally triggered by winterlike (long day) photoperiods and low ambient temperatures. Recent data demonstrate that intracerebroventricular injection of neuropeptide Y (NPY) reliably induces torpor like-hypothermia that resembles natural torpor in hamsters. NPY-induced torpor like-hypothermia is also produced by intracerebroventricular injections of an NPY Y1 receptor agonist but not by injections of an NPY Y5 receptor agonist. Both Y1 antagonist and Y5 antagonist injections significantly reduced food ingestion 24 h after treatment. They conclude that activation of NPY Y1 receptors is both sufficient and necessary for NPY-induced torpor-like hypothermia in hamsters.

First of all, we noticed that bezafibrate, a ligand of PPAR*α*, advances the active phase of locomotor behavior in wild type mice under 12h light-12h dark (LD) conditions [31]. Next, bezafibrate also advanced the phase in mice with lesions of the suprachiasmatic nucleus (SCN; the central clock in mammals) suggests that this phenomena depend on FEO. The circadian expression of clock genes such as period2, BMAL1, and Rev-erbalpha was also phase-advanced in various tissues (cortex, liver, and fat) without affecting the SCN. Bezafibrate also phase-advanced the activity phase that is delayed in model mice with delayed sleep phase syndrome (DSPS) due to a Clock gene mutation. The data suggests that PPAR*α* is a potential therapeutic target of drugs to treat circadian rhythm sleep disorders [31]. Furthermore, interestingly enough, Bezafibrate can advance the activity onset housing the mice under a long day (18 h of light and 6 h of darkness: LD 18 : 6) like summer, but not under a short day (LD 8 : 16) like winter [31]. These observations suggest that PPAR*α* is deeply involved in entrainment of the circadian clock to different seasonal change of LD conditions.

**Figure 2:**
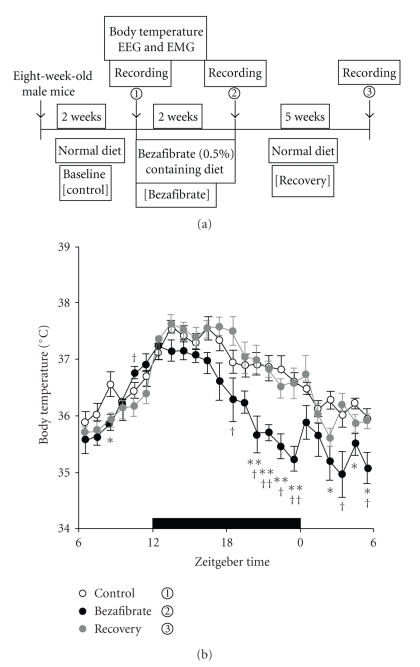
The effect of bezafibrate on body temperature (BT). (a) Time course (above) of BT in mice fed a control diet (open circles), a bezafibrate-containing diet (filled circles) or the control diet following recovery (gray circles). Revised from reference No.29.

Recently we also found out that bezafibrate-feeding alter sleep state and body temperature (BT) in laboratory mice [28]. Two-weeks feeding of bezafibrate decreased BT, especially during the latter half of the dark period which is very similar timing for torpor (Figure 2). BT rhythm and sleep/wake rhythm were phase advanced about 2-3 h by bezafibrate-feeding as observed in the phase advance of locomoter behavior. Bezafibrate-feeding also increased the electroencephalogram (EEG) delta-power in nonrapid eye movement (NREM) sleep compared with the control diet mice. Furthermore, bezafibrate-treated mice showed no rebound of EEG delta-power in NREM sleep after 6 h sleep deprivation, suggesting that sleep depth of bezafibrate-treated mice is very high as observed in hibernation animals. DNA microarray, and real-time RT-PCR analysis showed that bezafibrate-feeding increased levels of Neuropeptide Y(NPY) mRNA in the hypothalamus at both Zeitgeber time (ZT) 10 and ZT22, and decreased proopiomelanocortin-alpha mRNA in the hypothalamus at ZT10 in male ICR mice. Furthermore, the intracerebroventricular injection of antagonist for NPY Y1 receptor blocked torpor-like hypothermia, but not for EEG delta-power in NREM sleep in the mice [28]. As mentioned above, NPY is a key molecule for inducing torpor in the natural torpor animal, siberian hamsters. These findings demonstrate that PPAR*α* participate in the control of torpor through NPY production in the hypothalamus even in laboratory mice and suggest that activation of PPAR*α* enhance deep sleep and improve resistance to sleep loss through unknown mechanisms. But I do not exclude the possibility that PPAR*α* activated lipid metabolites and ketogenesis may have a role for torpor via unknown pathway. 

**Figure 3:**
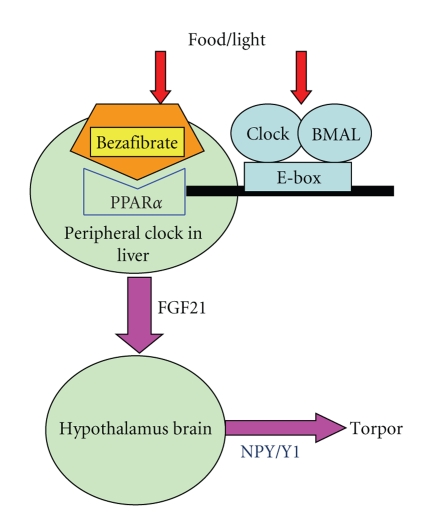
Role of PPAR*α* in the control of torpor through FGF21-NPY pathway. Environmental changes like food (fasting) and light enter the body from PPAR*α* and clock gene products, Clock/Bmal, respectively. Clock/Bmal regulated-rhythmic PPAR*α* expression. PPAR*α* directly transactivate FGF21 and FGF21 may enter brain and effect the expression of Neuropeptide Y(NPY). NPY can induce torper through Y1 receptor for NPY.

Thus, I would propose a new model for a role of PPAR*α* in the control of torpor through FGF21-NPY pathway (Figure 3). This model may partly explain the importance of circadian—and photoperiod—regulation of PPAR*α* to tell seasons in our body. This PPAR*α*-FGF21-NPY pathway might be a missing link between circadian clock and seasonal clock.
